# Crystal structure of human nucleosome core particle containing enzymatically introduced CpG methylation

**DOI:** 10.1002/2211-5463.12064

**Published:** 2016-05-03

**Authors:** Yoshifumi Fujii, Masatoshi Wakamori, Takashi Umehara, Shigeyuki Yokoyama

**Affiliations:** ^1^ RIKEN Systems and Structural Biology CenterTsurumiYokohamaJapan; ^2^ RIKEN Structural Biology LaboratoryTsurumiYokohamaJapan; ^3^ RIKEN Center for Life Science TechnologiesTsurumiYokohamaJapan; ^4^ PRESTO, Japan Science and Technology Agency (JST)KawaguchiSaitamaJapan

**Keywords:** chromatin, crystal structure, epigenetics, histone, transcription

## Abstract

Cytosine methylation, predominantly of the CpG sequence in vertebrates, is one of the major epigenetic modifications crucially involved in the control of gene expression. Due to the difficulty of reconstituting site‐specifically methylated nucleosomal DNA at crystallization quality, most structural analyses of CpG methylation have been performed using chemically synthesized oligonucleotides, There has been just one recent study of nucleosome core particles (NCPs) reconstituted with nonpalindromic human satellite 2‐derived DNAs. Through the preparation of a 146‐bp palindromic α‐satellite‐based nucleosomal DNA containing four CpG dinucleotide sequences and its enzymatic methylation and restriction, we reconstituted a ‘symmetric’ human CpG‐methylated nucleosome core particle (NCP). We solved the crystal structures of the CpG‐methylated and unmodified NCPs at 2.6 and 3.0 Å resolution, respectively. We observed the electron densities of two methyl groups, among the eight 5‐methylcytosines introduced in the CpG‐fully methylated NCP. There were no obvious structural differences between the CpG‐methylated ‘symmetric NCP’ and the unmodified NCP. The preparation of a crystallization‐grade CpG‐methylated NCP provides a platform for the analysis of CpG‐methyl reader and eraser proteins.

**Abbreviations**
5mC: 5‐methylcytosine
bp: base pair
BSA: bovine serum albumin
CBB: Coomassie Brilliant Blue
ds: double‐stranded
*E. coli*: *Escherichia coli*
EDTA: ethylenediaminetetraacetic acid
IPTG: isopropyl β‐d‐1‐thiogalactopyranoside
MBD: methyl CpG‐binding domain
MPD: 2‐methyl‐2,4‐pentanediol
NCP: nucleosome core particle
ORF: open reading frame
PAGE: polyacrylamide gel electrophoresis
RMSD: root‐mean‐square deviation
SAM: *S*‐adenosylmethionine
SHL: superhelical location
SRA domain: SET and RING finger associated domain

Eukaryotic DNA methylation is one of the key chromatin modifications that play crucial roles in the regulation of the epigenetic status 1. Cytosine methylation at the C5 position (5‐methylcytosine, 5mC) of CpG dinucleotides is common in eukaryotes. In vertebrates, methylation only occurs at this site. In the human genome, the CpG dinucleotides are clustered in short CpG‐rich DNA stretches called ‘CpG islands’ and regions with repetitive DNA sequences, such as pericentromeric satellite regions 2, 3. The CpG islands exist in over half of the human gene promoters 4 and are usually kept unmethylated during development and differentiation 2, whereas the methylation of the CpG islands generally leads to long‐term gene silencing. CpG island methylation is involved in a variety of biological phenomena, including X chromosome inactivation, genomic imprinting, genome stability, and embryogenesis 2, 3. Furthermore, aberrant CpG methylation is a characteristic observed in several human diseases, such as cancers 5, atherosclerosis 6, and schizophrenia 7.

The CpG methylation status is dynamically modulated during development, through a combination of active modification by cytosine methyltransferases and 5mC oxidases and passive dilution 8. The methylated CpG sites (i.e., 5′‐mCG‐3′/5′‐mCG‐3′ where mC indicates 5mC) are typically recognized by MBD (methyl CpG‐binding domain)‐containing proteins 9. The MBD proteins recruit additional proteins, such as histone deacetylases and chromatin remodeling factors, thereby leading to transcriptionally repressed chromatin. This connection between the DNA methylation state and the chromatin status is critically involved in the regulation of several diseases. As an example, the loss of MeCP2, one of the MBD proteins, is reportedly correlated with Rett syndrome 10, 11. In addition, MBD2 is known to mediate the transcriptional silencing of hypermethylated genes in cancer 12.

Biochemical CpG methylation, for the reconstitution of a CpG‐methylated nucleosome, has been performed, using one of the *de novo* CpG methyltransferases from *Spiroplasma*,* M.Sss*I 13, 14. For example, Choy *et al*. 15 reported the CpG methylation of 5S rDNA in a reconstituted nucleosome by *M.Sss*I. In addition, a nucleosome containing hemimethyl CpG was reconstituted, using nucleosome array DNA methylated by *M.Sss*I 16. Furthermore, Osakabe *et al*. 17 recently reconstituted nucleosome core particles (NCPs) containing *M.Sss*I‐methylated CpG dinucleotide sites, using a 160‐bp human satellite 2 DNA.

Although a variety of NCP structures have been solved thus far, the tertiary structure of the NCP containing CpG‐methylated DNA still remains poorly understood. This is probably due to the difficulty in reconstituting a crystallization quality CpG‐methylated NCP in a milligram‐scale quantity. Recently, the crystal structures of CpG‐methylated human satellite 2 NCPs were reported 17. The structures of two NCPs containing nonpalindromic DNAs with six CpG dinucleotides, derived from a 160‐bp human satellite 2 DNA, were solved. In the report, the overall structures of the CpG‐methylated and unmodified NCPs, both composed of one of the satellite 2‐derived DNAs, are essentially the same, and no structural effect of the CpG methylation on the NCP DNA was observed. As the NCPs composed of nonpalindromic DNAs might have been packed in opposite orientations in the crystals, the structure determination was not simple. In contrast, the structure determination is expected to be straightforward with a ‘symmetric’ CpG‐methylated NCP with a palindromic DNA. Therefore, in this study, we reconstituted human NCPs with a 146‐bp palindromic α‐satellite‐based DNA containing four methylated CpG sites, and examined whether a different CpG methylation pattern in a different DNA sequence of the NCP affects the nucleosomal structure. The CpG sites were enzymatically fully methylated, as examined by digestions using the methylation‐sensitive restriction endonuclease *Eco*72I and the methylation‐dependent restriction endonuclease *Msp*J1. We solved the crystal structure of the ‘symmetric’ CpG‐fully methylated NCP at 2.6 Å resolution, and compared it with that of the unmodified NCP solved at 3.0 Å. We confirmed that the CpG full methylation does not essentially affect the nucleosome structure, regardless of the base sequence of the DNA.

## Results

### Design for the preparation of CpG‐methylated nucleosomal DNA

**Figure 1:**
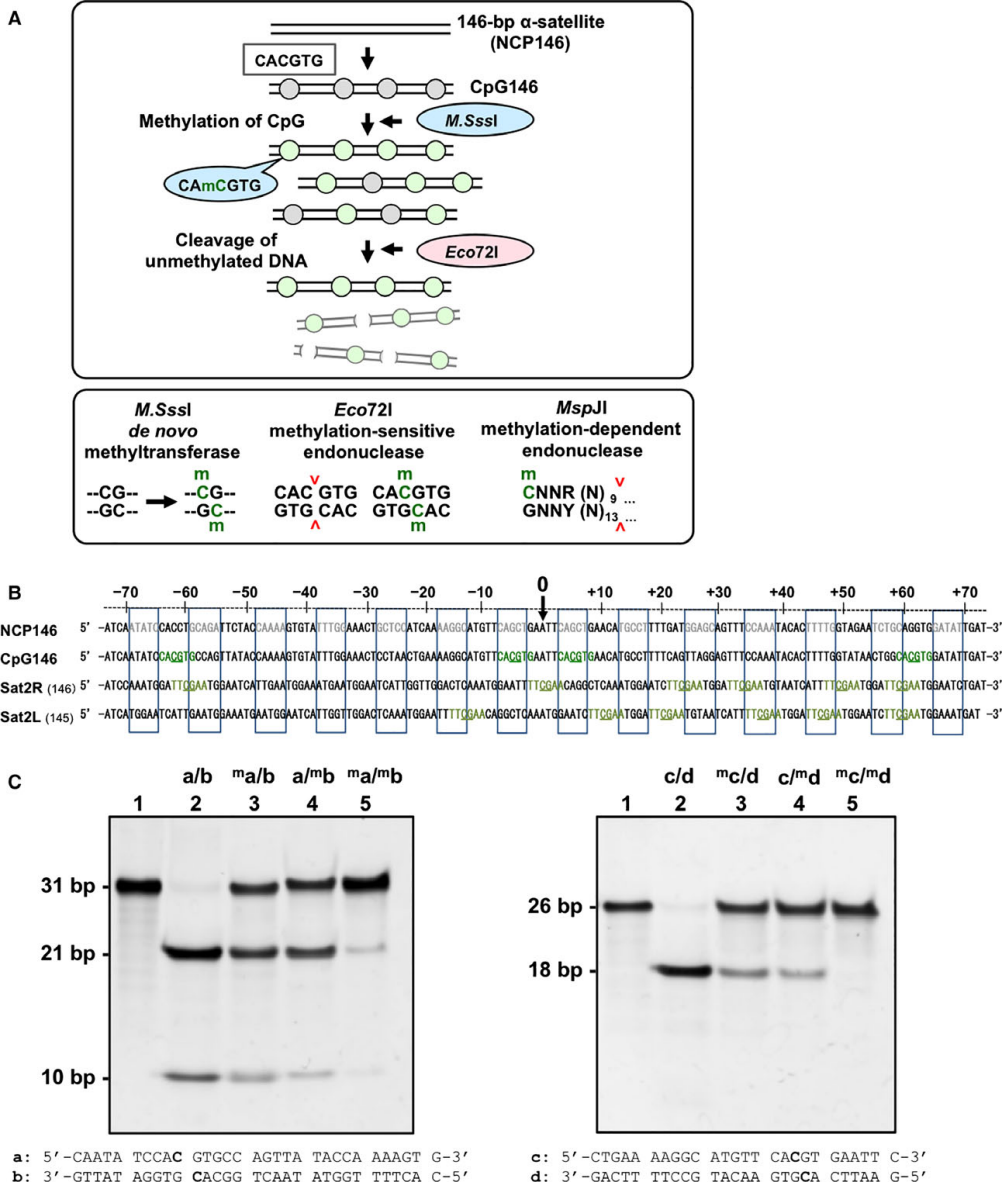
CpG methylation of nucleosomal DNA. (A) Scheme for the preparation of CpG‐methylated nucleosomal DNA. The 146‐bp human α‐satellite nucleosomal DNA 18 was mutated to contain four CpG dinucleotide‐containing CACGTG sequences, which are recognized by the methylation‐sensitive restriction enzyme *Eco72*I. The DNA designated as CpG146 is biochemically methylated by *M.Sss*I, and then digested by *Eco72*I, which is a CpG methylation‐sensitive restriction endonuclease for the CACGTG sequence. The full‐length 146‐bp nucleosomal DNA is methylated by *M.Sss*I, and a portion of the methylated DNA is examined by a digestion with *Eco*72I before the NCP reconstitution. (B) Sequences of nucleosomal DNAs used for crystal structure analyses. NCP146, 146‐bp α‐satellite nucleosomal DNA 18; and CpG146, CpG dinucleotide sequence‐introduced 146‐bp nucleosomal DNA (this study). Four CpG dinucleotide‐containing *Eco72*I recognition sequences, created by the site‐directed mutagenesis of NCP146, are shown in green. The positions of the CpG dinucleotides are underlined. Sat2R: the 145‐bp satellite 2 derivative right nucleosomal DNA 17. Sat2L: the 146‐bp satellite 2 derivative left nucleosomal DNA 17. The relative positions of DNA bases from the dyad axis (0) are indicated from −70 to +70 at the top. Minor groove‐inward facing regions, as reported by Chua *et al*. 31, are boxed within blue squares. The major grooves of the boxed DNA sequences are outward‐facing, and CpG‐methyl reader and/or eraser proteins can access 5mC. (C) *Eco72*I digestion patterns of double‐stranded CpG‐containing oligonucleotides. Oligonucleotides (a) and (b), or (c) and (d), which are both derived from the CpG146 sequence, were annealed to each other in the presence or absence of 5mC, at the indicated positions in bold letters. Lane 1, nondigested dsDNA; lanes 2–5, dsDNA digested with 1.8 units·pmol^−1^ *Eco72*I for 16 h. Lane 2, nonmethylated dsDNA; lanes 3 and 4, hemimethylated dsDNAs; and lane 5, fully methylated dsDNA. The superscript m at the top indicates a 5mC‐containing oligonucleotide. The DNA bands at 21‐bp and 10‐bp in the left panel and the DNA band at 18‐bp in the right panel indicate *Eco72*I‐digested DNA fragments.

To solve the crystal structure of a CpG‐methylated NCP, we first designed the construction of the 146‐bp α‐satellite‐based nucleosomal DNA (NCP146; 18) containing CpG dinucleotide sequences (Fig. 1A), because the α‐satellite sequence has a strong tendency for nuclesome formation, and is palindromic. Therefore, we regard it as one of the best nucleosomal DNAs for an X‐ray crystallographic analysis to examine whether the CpG methylation affects the nucleosomal structure. Since there is no CpG dinucleotide sequence in the original NCP146, we decided to introduce a couple of CpG dinucleotides into NCP146, at SHLs −1 and −6 in the half unit of the palindromic NCP146 sequence. For the positions, we selected one from a SHL near the DNA end (i.e., −1), and the other from a SHL near the axis (i.e., −6), to investigate the effects of CpG methylation on the NCP structure at different SHLs. Accordingly, the designed nucleosomal DNA, designated as CpG146, has four CpG dinucleotide sequences at around SHLs −6, −1, +1, and +6 (Fig. 1A,B).

To create the CpG sequence in CpG146, we introduced the CACGTG sequence, so the unmethylated nucleosomal DNA could be digested by the methylation‐sensitive restriction endonuclease *Eco72*I, after CpG methylation by the *de novo* CpG methyltransferase *M.Sss*I. This digestion method would allow us to purify the CpG146 nucleosomal DNA, in which four CpG dinucleotides are fully methylated, by its size, if *Eco72*I digests not only the CpG‐nonmethylated DNA but also the CpG‐hemimethylated DNA. We thus biochemically examined the ability of *Eco72*I to digest the non‐, hemi‐ and full‐methylated DNAs at its recognition sequence, CACGTG. As shown in Fig. 1C, the two CACGTG‐containing double‐stranded (ds) DNAs of CpG146 were almost completely resistant to the digestion by *Eco72*I at a concentration of 1.8 units·pmol^−1^ DNA, when both strands have 5mC modifications (i.e., full‐methyl DNA; lane 5). At this concentration, the nonmethylated (lane 2) and hemimethylated (lanes 3 and 4) DNAs were completely and partially digested by *Eco72*I, respectively. Therefore, we assumed that the purification of CpG‐fully methylated DNA is possible by the utilization of *Eco72*I at this concentration.

### Preparation of CpG146 DNA, *de novo* CpG methyltransferase, and CpG‐methylated CpG146 DNA

The designed CpG146 nucleosomal DNA was purified on a large‐scale, using the *E. coli* HB101 strain carrying the plasmid pWMD01 19, containing 16 tandem copies of the 73‐bp CpG146 unit sequence. From 10.8 L of the TB culture of this strain, we purified 95 mg of the plasmid DNA. The 73‐bp CpG146 unit DNA was excised from the plasmid, and self‐ligated to yield CpG146, essentially as previously described 20. Finally, 24 mg of the ligated CpG146 DNA was purified to near homogeneity and used for subsequent CpG methylation.

**Figure 2:**
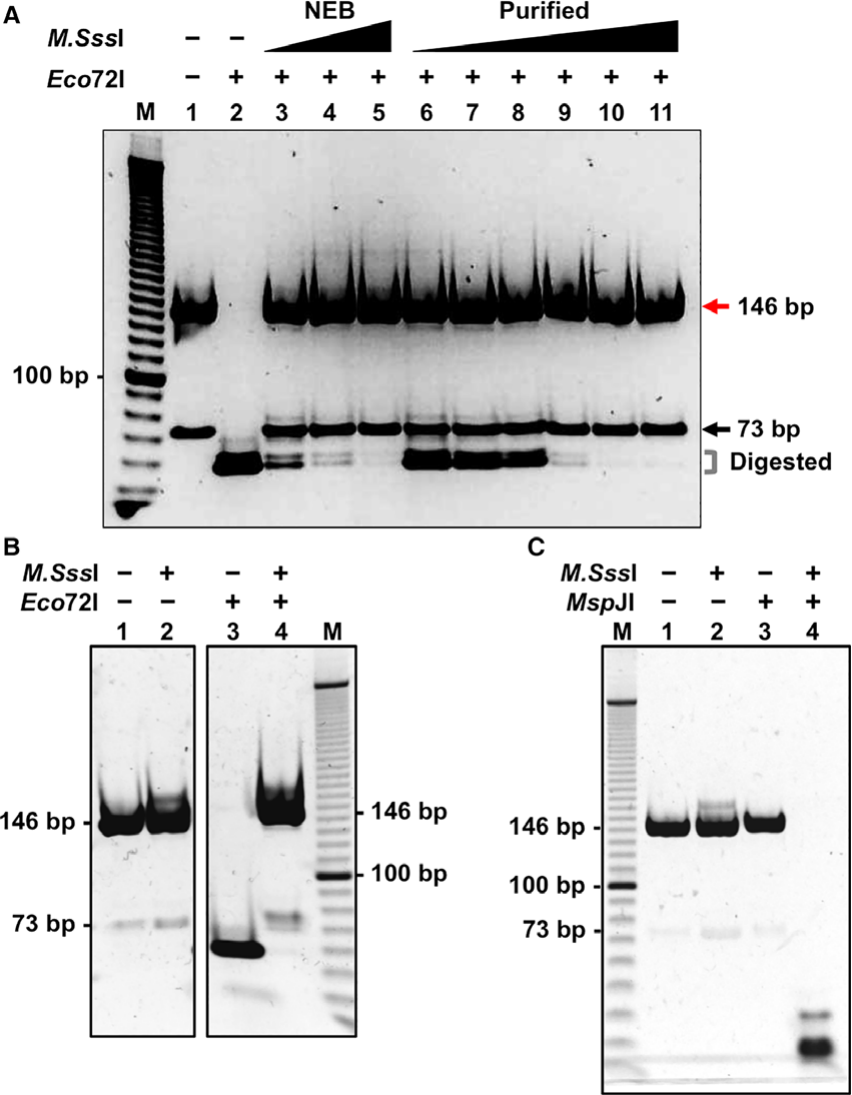
Biochemical methylation of CpG dinucleotide‐containing nucleosomal DNA (CpG146). Lanes are as follows: M, 10‐bp DNA ladder (Thermo Fisher Scientific, Waltham, MA, USA; cat. 10821‐015); (+), presence; and (−), absence of the respective enzyme shown on the left. (A) Comparison of the CpG methyltransferase *M.Sss*I enzymatic activities. CpG146 DNA was methylated with *M.Sss*I enzymes as follows: lanes 3–5, *M.Sss*I purchased from New England Biolabs (NEB, cat. M0226M); and lanes 6–11, the *M.Sss*I protein purified in this study. In lanes 1 and 2, CpG146 DNAs were incubated in the methylation reaction buffer, in the absence of *M.Sss*I. Each DNA sample was methylated with an increasing amount of *M.Sss*I, and the DNA from each reaction was purified, digested with the methyl‐sensitive restriction endonuclease *Eco72*I, and electrophoresed. The specific units of the NEB *M.Sss*I enzyme used in each reaction are as follows: lane 3, 2.0 units·μg^−1^ DNA; lane 4, 3.0 units·μg^−1^ DNA; and lane 5, 4.0 units·μg^−1^ DNA. The amount of the purified *M.Sss*I protein used in each reaction is as follows: lane 6, 0.032 μg·μg^−1^ DNA; lane 7, 0.063 μg·μg^−1^ DNA; lane 8, 0.13 μg·μg^−1^ DNA; lane 9, 0.25 μg·μg^−1^ DNA; lane 10, 0.50 μg·μg^−1^ DNA; and lane 11, 1.0 μg·μg^−1^ DNA. The positions of the 146‐bp CpG146 nucleosomal DNA, the half unit of CpG146, and the digested DNAs are shown by a red arrow, a black arrow, and a gray square bracket, respectively. (B) Confirmation of the CpG‐methylated CpG146 DNA by *Eco72*I digestion. The digestion was performed with 20 units·μg^−1^ DNA (1.8 units·pmol^−1^ DNA). Lanes 1 and 3, unmodified CpG146 DNA; lanes 2 and 4, *M.Sss*I‐treated CpG146 DNA. In lanes 3 and 4, the nucleosomal DNA was subsequently incubated with the methylation‐sensitive restriction enzyme *Eco72*I. (C) Confirmation of the CpG‐methylated CpG146 DNA by *Msp*JI digestion. The digestion was performed with 5 units·μg^−1^ DNA (0.45 units·pmol^−1^ DNA). Lanes 1 and 3, unmodified CpG146 DNA; lanes 2 and 4, *M.Sss*I‐treated CpG146 DNA. In lanes 3 and 4, the nucleosomal DNA was subsequently incubated with the methylation‐dependent restriction enzyme *Msp*JI.

To prepare a larger quantity of the methylated CpG146 DNA with quality suitable for crystallization, we chose the *Spiroplasma M.Sss*I enzyme as a *de novo* CpG methyltransferase. We subcloned the ORF of *M.Sss*I into the bacterial expression vector pET15b, for expression of the enzyme on a milligram‐scale. We purified approximately 8 mg of the *M.Sss*I enzyme, with a total of 96 000 units of enzymatic activity. The specific activity of the purified *M.Sss*I was calculated in comparison with the *M.Sss*I commercially available from New England Biolabs (NEB, Ipswich, MA, USA; cat. M0226M), by comparatively analyzing the *Eco72*I digestion efficiency toward the, respectively, methylated CpG146 DNA (Fig. 2A). One unit of the specific activity is defined by NEB as the amount of enzyme required to protect 1 μg of λ DNA, in a total reaction volume of 20 μL in 1 h at 37 °C, against cleavage by the *Bst*UI restriction endonuclease. Assuming that the amounts of digested DNA in lane 4 (3 units·μg^−1^ DNA; equivalent to 0.27 units·pmol^−1^ DNA) and lane 9 (0.25 μg·μg^−1^ DNA; equivalent to 0.023 μg·pmol^−1^ DNA) are nearly the same (i.e., bands shown by the gray square bracket), we calculated that our purified *M.Sss*I has a specific activity of 12 000 units·mg^−1^.

Using the purified *M.Sss*I enzyme, 6 mg of CpG146 nucleosomal DNA was methylated at the enzyme concentration of 6 units·μg^−1^ DNA, which is 0.54 units·pmol^−1^ DNA and two‐fold higher than the methylation conditions in lanes 4 and 9 in Fig. 2A. After the methylation reaction, we obtained 5.6 mg of the CpG146 nucleosomal DNA, by phenol–chloroform extraction. The yield of the DNA purification was 93%.

### Validation of the CpG full methylation of the CpG146 DNA

Using a portion of the methylated CpG146 DNA, we examined whether the DNA has CpG full methylation as designed, by the usage of two restriction endonucleases and one MBD protein. First, we used the *Eco72*I enzyme, which digests non‐ and hemimethylated DNA, but does not digest full‐methylated DNA (Fig. 2B). As expected, the digestion of the methylated CpG146 DNA by *Eco72*I was negligibly detected (lane 4) under the conditions, where the unmethylated CpG146 was completely digested (lane 3). The efficiency of the CpG146 DNA methylation was estimated to be more than 95%, by a densitometric analysis. We also validated the methylation of the CpG146 DNA, by using the methylation‐dependent restriction endonuclease, *Msp*JI (Fig. 2C). *Msp*JI digests DNA at the positions 9‐ and 13 bases after the sequence 5′‐mCNNR‐3′, where mC, N, and R indicate 5mC, A/G/C/T, and A/G, respectively. Thus, the four CG dinucleotide‐containing sequences around SHLs −6, −1, +1, and +6 in CpG146 can be digested by *Msp*JI, when the DNA is CpG‐methylated (Fig. 1). As expected, the methylated CpG146 DNA was completely digested by *Msp*JI (lane 4 in Fig. 2C), whereas the unmodified CpG146 DNA remained undigested (lane 3). As judged from the digestion assays, we concluded that the purified CpG146 DNA was fully CpG methylated.

**Figure 3:**
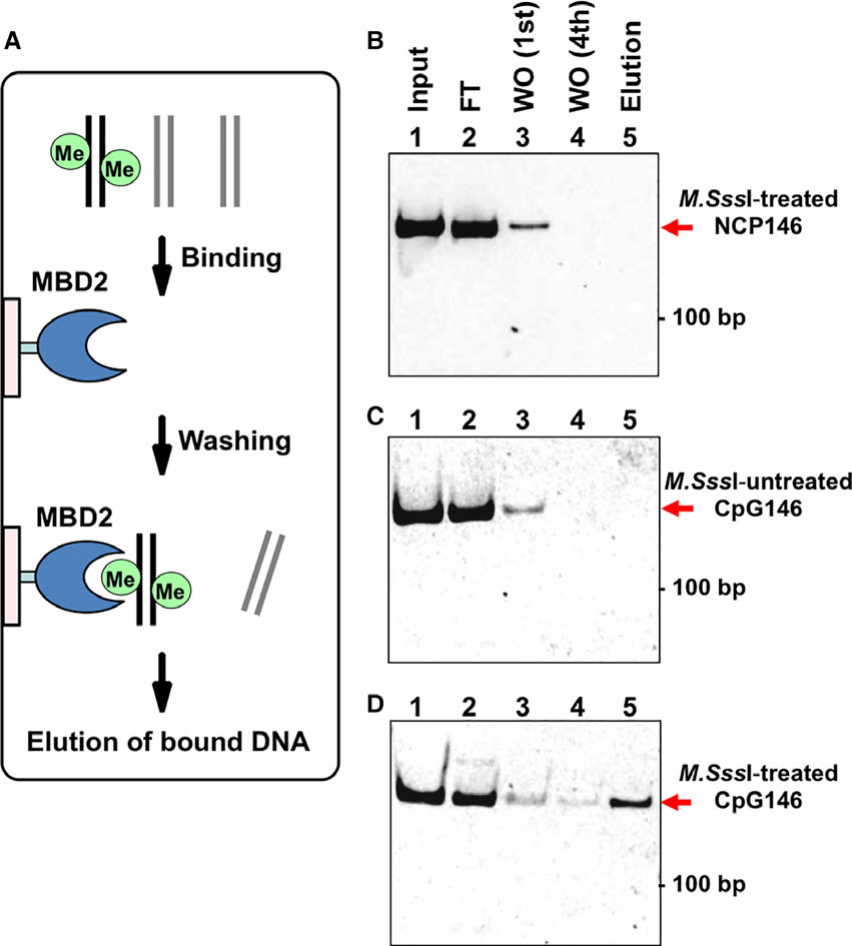
Binding analysis between MBD2 and nucleosomal DNAs. (A) Scheme of the binding analysis. (B–D) Results of the MBD2‐binding analysis. The nucleosomal DNAs used in the assay are as follows: (B) *M.Sss*I‐treated146‐bp α‐satellite DNA (NCP146); (C) *M.Sss*I‐untreated 146‐bp α‐satellite‐based DNA containing four CpG sites (CpG146); and (D) *M.Sss*I‐treated CpG146 DNA. Lane 1, Input nucleosomal DNA (125 ng); lane 2, flow‐through fraction after the incubation of nucleosomal DNA with immobilized MBD2; lane 3, wash fraction of the first washing step; lane 4, wash fraction of the fourth washing step; lane 5, eluted fraction. In each panel, the position of the 100‐bp DNA is indicated on the right, and the position of the nucleosomal DNA is indicated by a red arrow.

Next, we examined whether the purified CpG146 DNA with the CpG full methylation is biochemically functional, by assessing the DNA‐binding ability of MBD2 (Fig. 3A). MBD2 is one of the MBD‐containing proteins that specifically recognize the full‐methyl CpG sequence, and it shows higher affinity for methylated DNA than other MBD domains 21. We examined MBD2 binding to three nucleosomal DNAs: *M.Sss*I‐treated NCP146 DNA containing no CpG dinucleotide site, *M.Sss*I‐untreated CpG146 DNA containing four CpG dinucleotide sites, and *M.Sss*I‐treated CpG146 DNA. MBD2 did not bind to either the *M.Sss*I‐treated NCP146 DNA (lane 5 in Fig. 3B) or the *M.Sss*I‐untreated CpG146 DNA (lane 5 in Fig. 3C), as expected from their lack of CpG sites and CpG methylation, respectively. In contrast, MBD2 bound to the *M.Sss*I‐treated CpG146 DNA (lane 5 in Fig. 3D), validating the biochemical functionality of the *M.Sss*I‐treated, CpG‐methylated CpG146 DNA.

### Reconstitution and crystallization of CpG‐methylated NCP

**Figure 4:**
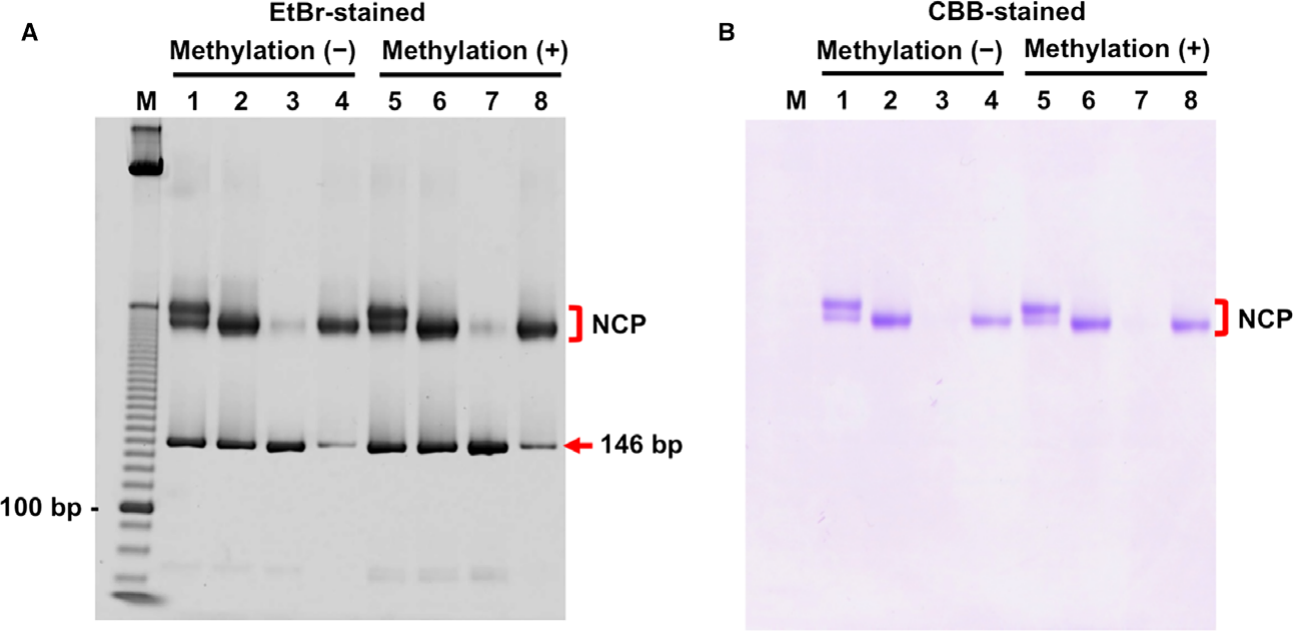
Native PAGE of the NCP samples used for crystallization. The positions of the NCPs and the free CpG146 DNAs are shown by red square brackets and a red arrow, respectively. (A) Ethidium bromide‐stained gel. (B) CBB‐stained gel. Lane M, 10‐bp DNA ladder (Thermo Fisher Scientific; cat. 10821‐015). Lanes 1–4, nucleosome core particles containing CpG146 DNA untreated with CpG methyltransferase *M.Sss*I. Lanes 5–8, nucleosome core particles containing CpG146 DNA treated with *M.Sss*I. Lanes 1 and 5, NCP samples after reconstitution and dialysis. Lanes 2 and 6, NCP samples after heat treatment. Lanes 3 and 7, supernatant fractions of MgCl_2_‐treated NCP samples. Lanes 4 and 8, precipitated fractions of the MgCl_2_‐treated NCP samples that were used for crystallization.

To understand the influence of CpG methylation on the nucleosome structure, we reconstituted the NCP using the CpG‐fully methylated CpG146 DNA and the four human core histone proteins, H2A, H2B, H3, and H4, essentially as previously described 19, 20. For a structural comparison, we also reconstituted the NCP using the unmodified CpG146 DNA. We found that the CpG‐methylated NCP (lane 5 in Fig. 4A,B) was reconstituted apparently as efficiently as the unmodified NCP (lane 1 in Fig. 4A,B), as shown by the ethidium bromide‐stained (i.e., DNA‐stained; Fig. 4A) and CBB‐stained (i.e., protein‐stained; Fig. 4B) native PAGE images. The reconstituted NCPs were then subjected to heat shifting (lanes 2 and 6 in Fig. 4A,B), and the free nucleosomal DNAs were removed by the magnesium‐dependent condensation of the NCPs (lanes 3 and 7 in Fig. 4A). The purified NCP samples, which are predominantly composed of NCPs with a minor portion of free nucleosomal DNAs (lanes 4 and 8 in Fig. 4A), were subjected to crystallization.

**Figure 5:**
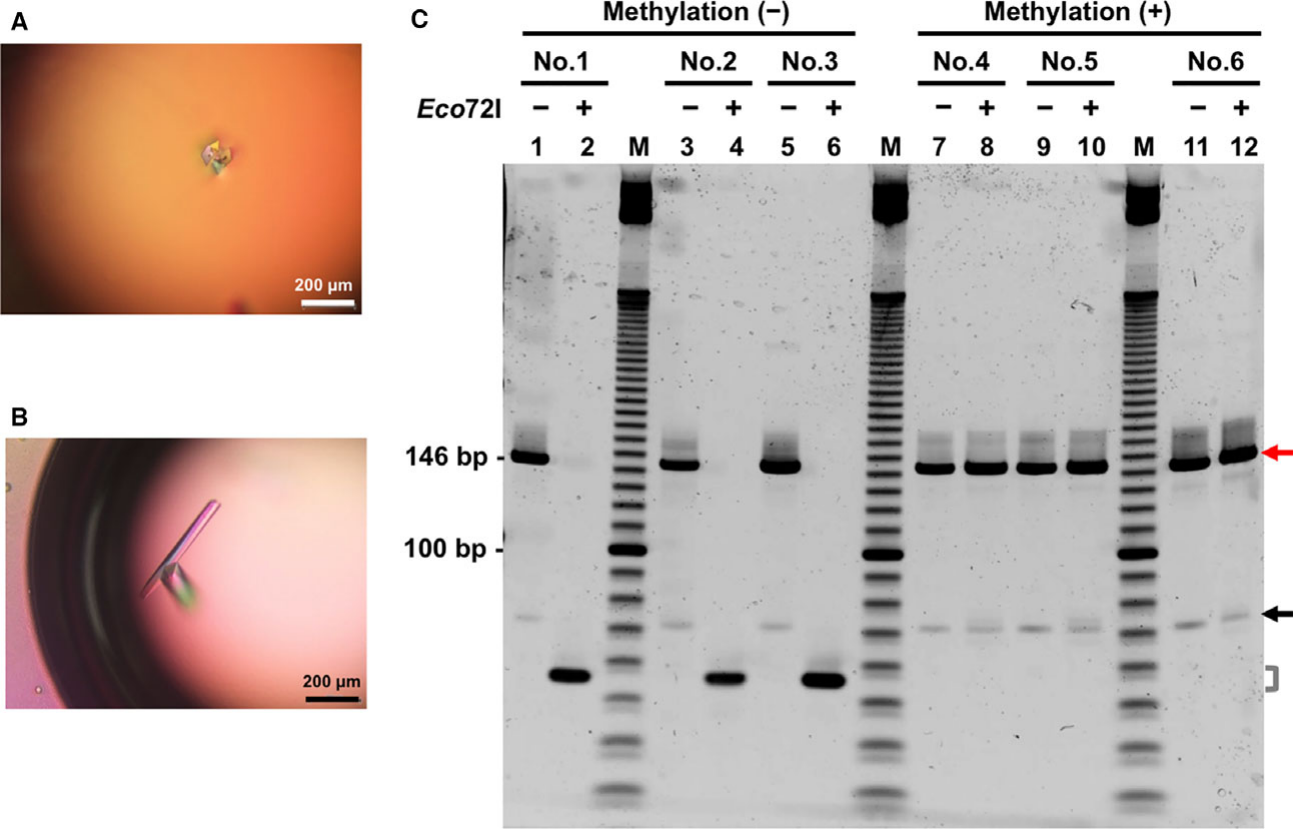
Crystallization of the CpG‐methylated CpG146 NCP. (A) Crystals of the CpG‐methylated CpG146 NCP. Diffraction data were collected to 2.6 Å resolution, using one of the crystals. (B) Crystals of the unmethylated CpG146 NCP. Diffraction data were collected to 3.0 Å resolution, using one of the crystals. (C) Confirmation of the CpG methylation of the crystallization droplets. *Eco72*I‐digestion patterns of the nucleosomal DNAs extracted from three different crystal‐yielding droplets of unmodified (Nos. 1–3) and CpG‐methylated CpG146 NCPs (Nos. 4–6) are shown. Lane M, 10‐bp DNA ladder (Thermo Fisher Scientific; cat. 10821‐015). The positions of the 146‐bp nucleosomal DNA and the half unit 73‐bp DNA are indicated by a red arrow and a black arrow, respectively. The position of the *Eco72*I‐digested 56‐bp fragment is indicated by a gray square bracket.

We obtained crystals from both the CpG‐full‐methylated (Fig. 5A) and unmodified (Fig. 5B) CpG146 NCPs, under similar crystallization conditions. From the single crystals of the CpG‐methylated and unmodified CpG146 NCPs, we collected X‐ray diffraction data sets at BL32XU of SPring‐8. To exclude the possibility that the methylated CpG sites were demethylated or degraded during the crystallization, we collected crystallization droplets that yielded crystals of the NCPs, and examined them by the *Eco72*I digestion to determine whether the designed CpG methylation remained intact in the NCP. From three crystallization droplets of both the CpG‐methylated and unmodified CpG146 NCPs, we extracted the nucleosomal DNAs by proteinase K treatment and phenol–chloroform extraction, and digested them with *Eco72*I (Fig. 5C). We confirmed that the CpG full methylation in the CpG‐methylated NCPs remained intact for several months after the crystallization (Fig. 5C).

### Overall structures of CpG‐methylated and unmodified NCPs

**Figure 6:**
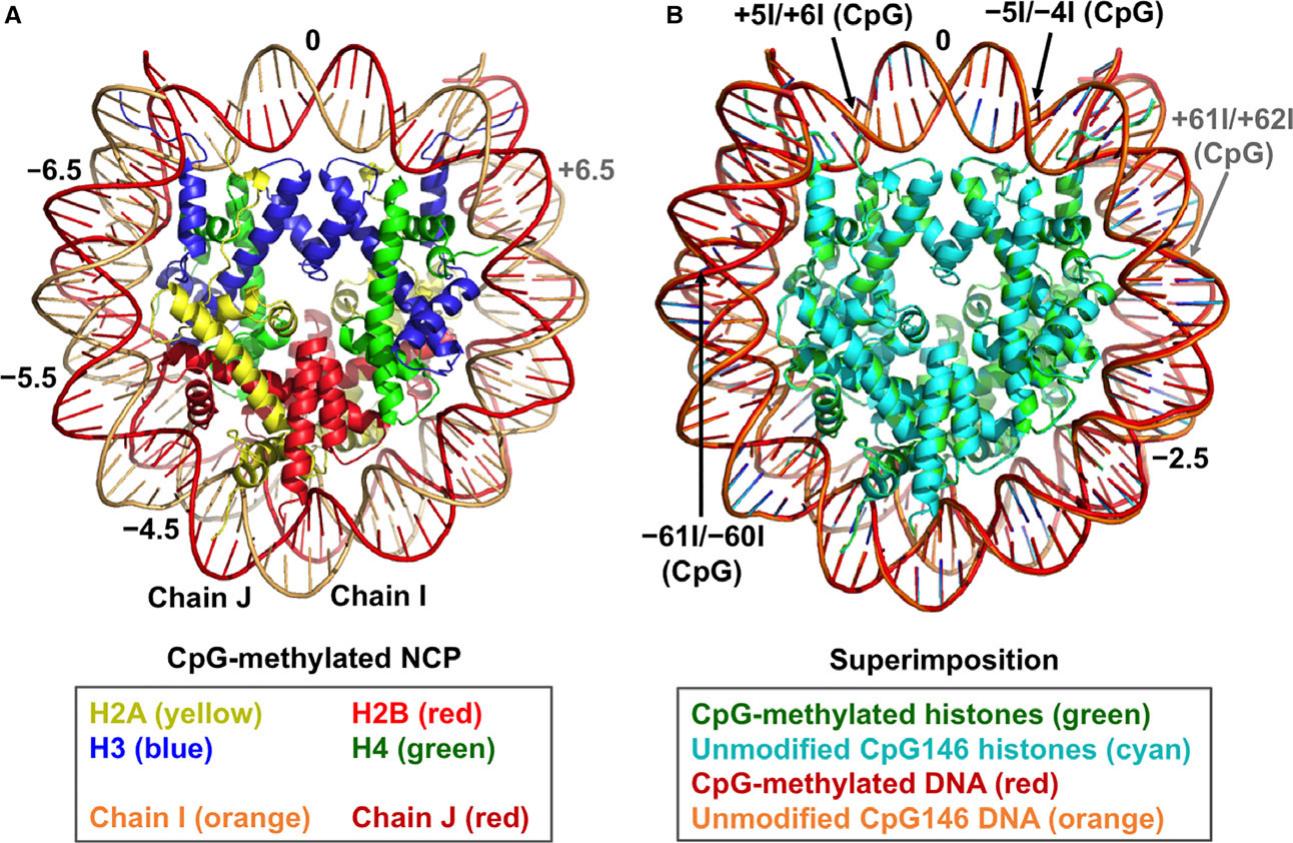
Overall structure of the CpG‐methylated NCP. (A) Ribbon diagram structure of CpG‐methylated CpG146 NCP. Color codes for histones and DNAs are indicated at the bottom. SHL −6.5, located behind the first turn of the DNA duplex, is denoted in gray. (B) Superimposition of the CpG‐methylated and unmethylated CpG146 structures. Color codes for histones and nucleosomal DNAs are indicated at the bottom. The positions of the four CpG dinucleotide sites are shown by arrows. The +61I/+62I CpG dinucleotide, located behind the first turn of the DNA duplex, is denoted in gray.

The crystal structures of the CpG‐methylated and unmodified NCPs were refined to 2.6 and 3.0 Å resolution, respectively, by molecular replacement as described in the Materials and methods. To exclude model bias, we used the auto‐modeling program AutoBuild (PHENIX; www.phenix-online.org). Consequently, the *R*_work_/*R*_free_ decreased by several percentage points. The final refinement statistics are summarized in Table 1. We solved the structures of the CpG‐methylated and unmodified CpG146 NCPs (Fig. 6A,B). In the electron density map, both ends of the 146‐bp DNA were clearly observed in the CpG‐methylated NCP (data not shown), as also observed for the unmodified NCP in the present and previous studies 22, 23 using the 146‐bp DNA. The histone‐fold core regions were also clearly visible in both NCPs (Fig. 6A,B). The two overall NCP structures were nearly identical, as demonstrated by superimposition (Fig. 6B). In the superimposition of the atomic models of the CpG‐methylated and unmodified NCP structures, the global RMSD (root‐mean‐square deviation) values of all of the protein Cα atoms and the DNA sugar–phosphate backbones were as small as 0.27 and 0.46 Å, respectively. Between the CpG‐methylated NCP and the modeled NCP146 (PDB: 1KX3) 22 structures, the RMSD values of all of the Cα atoms and the DNA sugar–phosphate backbone were 0.35 and 0.93 Å, respectively.

**Table 1:** Data collection and refinement statistics

	CpG‐methylated CpG146 NCP	Unmodified CpG146 NCP
Wavelength (Å)	1.0	1.0
Resolution range (Å)	20.00–2.60 (2.69–2.60)^a^	20.00–3.00 (3.11–3.00)
Space group	*P* 2_1_ 2_1_ 2_1_	*P* 2_1_ 2_1_ 2_1_
Unit cell dimensions (*a*,* b*,* c*) (Å)	106.54, 109.67, 181.35	106.50, 109.05, 177.02
Angles (α, β, γ) (°)	90, 90, 90	90, 90, 90
Total reflections	224 900 (19 499)	199 295 (18 140)
Unique reflections	64 673 (6481)	41 785 (3451)
Multiplicity	3.5 (3.1)	4.8 (4.4)
Completeness (%)	99.52 (99.88)	97.91 (86.52)
Mean *I*/σ(*I*)	5.56 (1.45)	4.87 (0.68)
Wilson *B*‐factor	64.33	109.59
*R*_merge_	0.113 (0.95)	0.145 (1.77)
*R*_meas_	0.132 (1.14)	0.163 (2.01)
CC_1/2_ b	0.99 (0.58)	0.99 (0.62)
CC*^c^	0.99 (0.86)	0.99 (0.88)
*R*_work_	0.197 (0.310)	0.202 (0.389)
*R*_free_	0.252 (0.365)	0.259 (0.423)
Number of non‐hydrogen atoms	12 171	12 081
Macromolecules	12 085	12 077
Ligands	7	4
Water molecules	79	0
Protein residues	768	767
RMSD (bonds) (Å)	0.004	0.003
RMSD (angles) (°)	0.65	0.58
Ramachandran favored (%)	98	98
Ramachandran allowed (%)	1.3	2.1
Ramachandran outliers (%)	0.27	0.27
Clash score	5.22	4.63
Average *B*‐factor (Å^2^)	96.4	146.8
Macromolecules	96.6	146.8
Ligands	116.5	167.7
Solvent	60.4	
PDB ID	5B2J	5B2I

^a^ Values in parentheses are for highest resolution shell. ^b^CC_1/2_, percentage of correlation between intensities from random half datasets 41. ^c^CC*, Calculated from CC_1/2_.

### Electron density map around CpG‐methylated DNA regions

**Figure 7:**
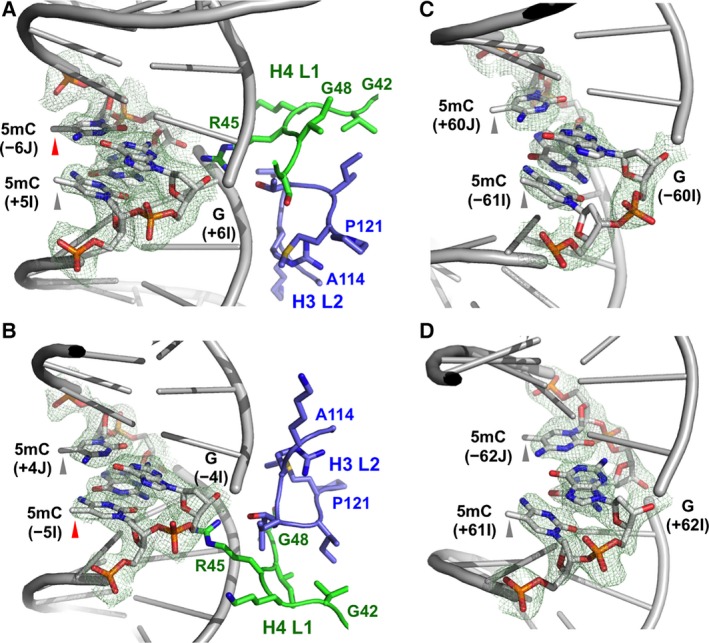
Electron density maps around the four methyl CpG dinucleotide sites. Meshes indicate the *2mF*_*o*_–*DF*_*c*_ electron density maps corresponding to the methylated CpG (5′‐mCG‐3′/5′‐mCG‐3′) sites. All maps are contoured at 1.0 σ. (A) mCG/mCG at +5/−6 and +6/−5 of chain I/J. (B) mCG/mCG at −5/+4 and −4/+5 of chain I/J. (C) mCG/mCG at −61/+60 and −60/+61 of chain I/J. (D) mCG/mCG at +61/−62 and +62/−61 of chain I/J. In each panel, the positions of the 5mC methyl groups are shown by arrowheads. Methyl groups with observed and unobserved electron densities are indicated by red and gray arrowheads, respectively. In (A) and (B), the H3 loop L2 of residues 114–121 (blue) and the H4 loop L1 of residues 42–48 (green), which both face toward the inside of the minor groove DNA, are shown by stick models.

There are four CpG‐fully methylated dinucleotide sites (i.e., 5′‐mCG‐3′/5′‐mCG‐3′), containing eight CpG‐methyl groups, in the present CpG‐methylated NCP structure (Fig. 1B). The *2mF*_*o*_–*DF*_*c*_ electron density maps with stick models are shown for the 5mCs at positions −61, −5, +5, and +61 of chain I and the 5mCs at −62, −6, +4, and +60 of chain J (Fig. 7A–D). In the *2mF*_*o*_–*DF*_*c*_ electron density maps, we observed the methyl groups of 5mC at positions −5 of chain I (Fig. 7B) and −6 of chain J (Fig. 7A), among the eight 5mC positions. The electron densities for the 5mC methyl groups were not observed at the other six positions, at −61, +5, and +61 of chain I and −62, +4, and +60 of chain J (Fig. 7A–D). In general, it is difficult to distinguish the existence of one methyl group, which is located in a DNA region with high *B*‐factors, from the 2*mF*_*o*_ − *DF*_*c*_ electron density map at 2.6 Å resolution in the NCP structures. We placed all eight 5mC methyl groups in the final coordinates of the CpG‐methylated CpG146 NCP.

### Local structural differences between the CpG‐full‐methylated and unmodified NCPs

**Figure 8:**
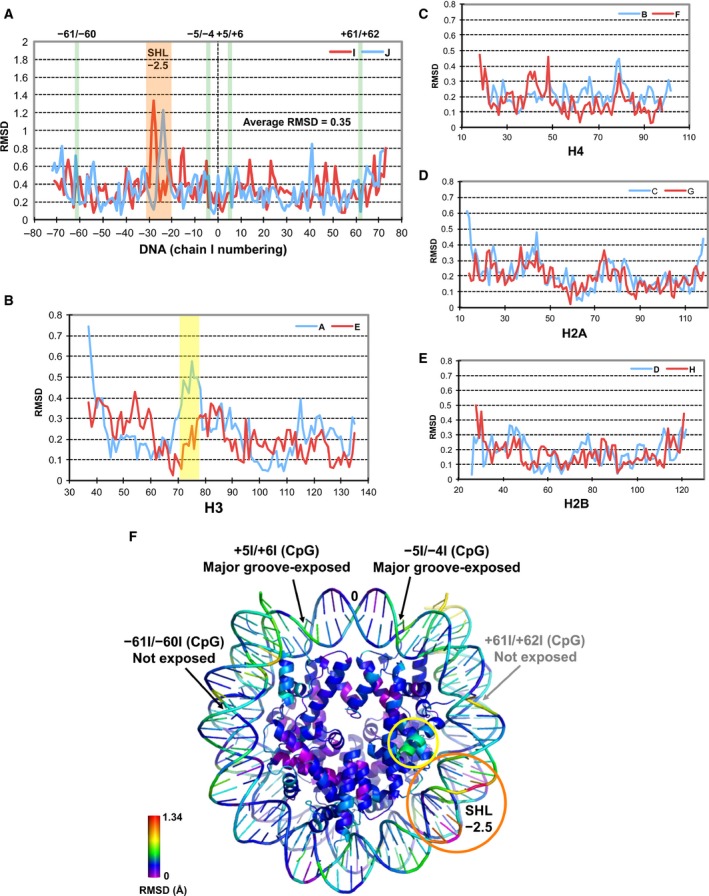
RMSD plots between CpG‐methylated and unmodified CpG146 NCPs. The numbers of nucleotides in the nucleosomal DNAs (A), or amino acids of the histones (B to E), are indicated on the X‐axes. RMSD value is shown on the *Y*‐axis. Colors for DNA chains I and J (A) or histones A to H (B to E) are indicated at the top‐right corners. (A) RMSD plots of overall DNA chains. The sequence numbers of the nucleotides are shown by the chain I numbering. The four positions of the CpG dinucleotide sites are shown in green. The region around SHL −2.5, with the most prominent RMSD difference at −28I/+24J, is shown in orange. (B to E) RMSD plots of core histone proteins. (B) Histone H3. (C) Histone H4. (D) Histone H2A. (E) Histone H2B. In (B), the region showing the most prominent RMSD difference, observed around the α1 helix of the molecule‐A histone H3 (residues 70–78 of 64–78), is shown in yellow. (F) Representation of the RMSD difference between the two structures on the NCP structure. The RMSD difference between the CpG‐methylated and unmodified CpG146 NCPs is colored on the CpG‐methylated NCP structure. The color bar represents the RMSD difference from 0 Å^2^ (bottom) to 1.34 Å^2^ (top). The regions with the most prominent RMSD difference in the nucleosomal DNA and histone H3 are shown by the orange and yellow circles, respectively. The positions of the four CpG dinucleotide sites are shown by arrows. The +61I/+62I CpG dinucleotide, located behind the first turn of the DNA duplex, is denoted in gray. The major groove‐exposed or ‐unexposed positions of the methylated CpG dinucleotides are shown.

The overall structures of the CpG‐methylated and unmodified NCPs are basically the same. To examine if there are any local structural differences, we plotted the RMSD differences in all of the nucleotides along the nucleosomal DNA chains (Fig. 8A) and all of the modeled amino acid residues in the histone proteins (Fig. 8B–E). These RMSD differences are mapped onto the CpG‐methylated NCP structure, with color coding according to the RMSD differences (Fig. 8F). In the nucleosomal DNA, the most prominent RMSD difference is observed around SHL −2.5, with the peaks of −28I and +24J (Fig. 8A,F). The positions of the backbone phosphate groups of the four CpG‐methylated dinucleotide sites (i.e., −61/+60, −5/+4, +5/−6, and +61/−62 in chains I/J) are basically the same (Fig. 6B), and the differences in their RMSDs between the two NCPs are not significant (Fig. 8A,F). In the histone proteins, the structures of histones H2A, H2B, and H4 are basically the same, and they do not prominently deviate between the two NCPs (Figs 6B and 8B–F). In histone H3, the largest RMSD difference is observed around the α1 helix (residues 70–78 of 64–78) of molecule A (Fig. 8B,F), where the α1 helix of histone H3 and SHL −2.5 of the DNA are closer to each other in the nucleosome (Fig. 8F).

## Discussion

### Reconstitution of the CpG‐fully methylated NCP

Cytosine methylation of CpG dinucleotides in eukaryotes is one of the key epigenetic modifications regulating a variety of biological phenomena, such as gene silencing, genomic imprinting, and embryogenesis. From both biological and structural points of view, all of the possible statuses of CpG methylation, full‐, hemi‐, and nonmethyl CpG, are critically involved in the control of these phenomena. The difference in the methylation statuses is precisely recognized by a series of DNA‐binding domains, such as the MBD domain (full‐methyl CpG reader) 24, the SRA domain (hemi‐methyl CpG reader) 25, 26, 27, and the CXXC domain (nonmethyl CpG reader) 28, which subsequently trigger different biochemical reactions. Therefore, the precise reconstitution of a nucleosome with a designed pattern of CpG methylation, strictly discriminating full‐methyl from hemimethyl CpG and *vice versa*, was eagerly awaited for biochemical and structural studies in epigenetics and chromatin biology. Here, we biochemically addressed this issue, and obtained the crystallization‐grade NCP containing four CpG‐fully methylated dinucleotide sites.

As for the restriction specificity of *Eco*72I and its isozymes recognizing the CACGTG sequence (Fig. 1A), the methylation‐sensitivity of *Bbr*PI, which is one of the isozymes, is validated using 5mC‐substituted DNA 29 or *M.Sss*I‐methylated DNA 30. However, it is unclear whether *Eco*72I or its isozyme is sensitive to the hemimethylation of the CpG dinucleotide in the CACGTG sequence. In this study, we found that *Eco*72I can digest the CpG‐hemimethylated CACGTG sequence about half as efficiently as the CpG‐nonmethylated one (Fig. 1C). Therefore, the present method enabled us to prepare a nucleosome with designed CpG full methylation, using a portion of the methylated nucleosomal DNA and *Eco*72I (Fig. 2B), which will be applicable to the preparation of NCPs with CpG‐fully methylated CACGTG sites.

### Structural comparison between the CpG‐full‐methyl and unmodified NCPs

We designed and prepared a 146‐bp α‐satellite‐based DNA containing four CpG dinucleotide sequences (CpG146), using enzymatic methylation and digestion, and reconstituted human NCPs with site‐specific CpG full methylation. Furthermore, we have solved the crystal structures of the CpG‐methylated and unmodified NCPs at 2.6 and 3.0 Å resolution, respectively. We found that the overall structure of the CpG‐methylated NCP is basically the same as that of the unmodified NCP (Fig. 6B). The CpG full methylation, verified by the digestion with two different endonucleases (Fig. 2B,C), does not affect the overall structure of the histone octamer and the conformations of the DNA in the NCP. We observed the electron densities corresponding to the 5mC methyl groups at two positions among the eight 5mC positions: the −6 5mC of chain J (SHL +0.5; Fig. 7A) and the −5 5mC of chain I (SHL −0.5; Fig. 7B). The two CpG dinucleotide sites are both located within the minor groove‐inward center, which is stabilized by the DNA‐binding loops of histones (i.e., L2 of histone H3 and L1 of histone H4) 31. The other two CpG dinucleotide sites (i.e., −61/+60 and +61/−62 in chains I/J) are not located at such histone‐stabilized DNA regions, and no clear electron densities for their 5mC methyl groups were observed (Fig. 7C,D). Similarly, the electron densities of the thymine methyl groups were observed for 30 among the 90 thymidine positions in the 2.6 Å CpG‐methylated NCP structure. Therefore, the partial observation of the 5mC methyl groups is reasonable, considering the 2.6 Å resolution. In this context, no electron density was observed for the 5mC positions in the previous CpG‐methylated NCP structures 17.

### The effect of the CpG full methylation on the NCP structure

In spite of the very small chemical change, the addition of the methyl group to the C5 position of cytosine can influence the major groove readout, using hydrophobic contacts and shape readout in the minor groove 32. Do the eight CpG methylations affect the local structures of the DNA and the histone octamer? The positions of the four CpG‐methylated dinucleotide sites (i.e., −61/+60, −5/+4, +5/−6, and +61/−62 in chains I/J) are shown in Fig. 8A. From the RMSD plots between the CpG‐methylated and the unmodified NCPs, the most prominent difference is observed around SHL −2.5, with peaks at −28I/+24J (Fig. 8A,F). This shift may cause a structural change around the nearby α1 helix of the molecule‐A histone H3. All four CpG dinucleotide sites (i.e., −61/+60, −5/+4, +5/−6, and +61/−62 in chains I/J) are far away from SHL −2.5 (Fig. 8F), and SHL −2.5 is located around one of the most flexible SHLs in this crystal packing 31, 33. Therefore, we conclude that the slight local structural shift between the CpG‐methylated and unmodified NCPs is basically attributable to variations in the analyzed crystals.

### Comparison with the CpG‐methylated human satellite 2 NCPs

**Figure 9:**
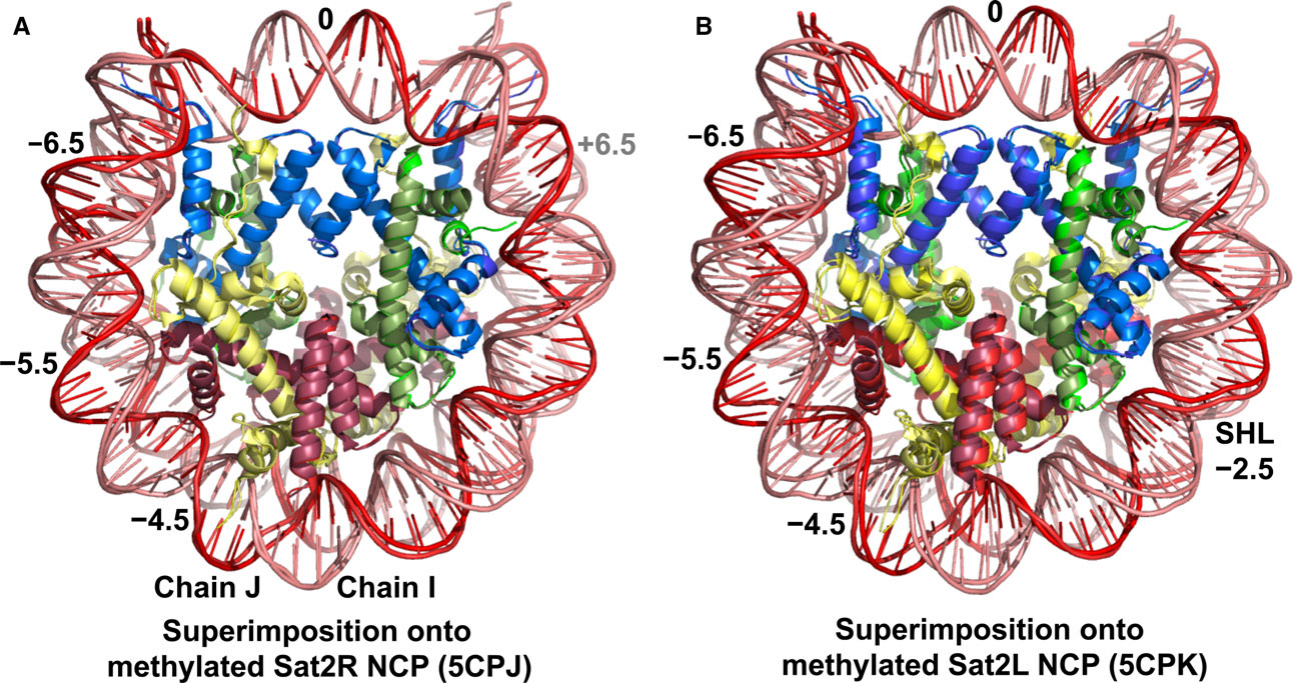
Structural comparison with the CpG‐methylated Sat2R and Sat2L NCPs. (A) Superimposition of the CpG‐methylated CpG146 and the Sat2R (PDB: 5CPJ) NCPs. (B) Superimposition of the CpG‐methylated CpG146 and the Sat2L (PDB: 5CPK) NCPs.

The present CpG‐methylated NCP structure was compared with the recently reported structures, at 2.63 and 3.15 Å resolution, of the human satellite 2 NCPs prepared with human nonpalindromic satellite 2 derivative sequences (i.e., Sat2R and Sat2L) (PDB IDs: 5CPJ and 5CPK) 17. Between our structure and the Sat2R and Sat2L NCP structures, the RMSD values of all Cα atoms and the DNA sugar–phosphate backbone are 0.48/0.38 and 1.98/1.94 Å, respectively. As shown in Fig. 9A,B, the histone octamers fit well, even though the nucleosomal DNAs do not. This is probably because the DNA sequences of CpG146 and Sat2R/L are quite different. Therefore, it has now been clarified that the CpG methylation does not detectably affect the NCP structure, regardless of the DNA sequence.

### Perspective for structural analysis of NCP complexes

The present methodology for the preparation of crystallization quality CpG‐fully methylated NCPs can facilitate structural analyses, such as that of a CpG‐methyl‐containing NCP in complex with MBD proteins 9. Two major groove‐exposed regions (i.e., −5/+4 and +5/−6 of chains I/J; see Figs 1B and 8F) among the four CpG dinucleotide sites in the CpG146 sequence are potential binding sites for a CpG‐binding protein, such as an MBD protein. Since the current crystal packing cannot accommodate the CpG‐methylated NCP in complex with an MBD domain, different crystallization conditions with new crystal packing will be required for the structural analysis of the MBD‐bound, CpG‐methylated NCP complex. The present methodology will be useful to understand whether and how the CpG methylation of nucleosomes, and the binding of CpG‐methyl reader and/or eraser proteins, influences nucleosomal assembly, positioning, and remodeling.

## Conclusions

Through the preparation of a 146‐bp α‐satellite‐based palindromic nucleosomal DNA, in which four CpG sequences are generated, and its enzymatic methylation and restriction, we reconstituted a ‘symmetric’ human CpG‐full‐methylated nucleosome core particle (NCP), and solved the crystal structures of the CpG‐methylated and unmodified NCPs at 2.6 and 3.0 Å resolution, respectively. The electron densities for two methyl groups among the eight 5mCs were observed in the CpG‐methylated NCP structure. The CpG‐fully methylated NCP resembled the unmodified NCP, except for the local shift in the region composed of the DNA around SHL −2.5 and the α1 helix of the molecule‐A histone H3, which are both far from the four CpG‐methylated sites. The present methodology to prepare a crystallization‐grade, CpG‐fully methylated NCP will facilitate future structural analyses of NCPs in complexes with CpG‐methyl reader and eraser proteins.

## Materials and methods

### Construction of CpG dinucleotide‐containing nucleosomal DNA

The nucleotide sequence of the 146‐bp palindromic DNA fragment of human α‐satellite DNA region, a kind gift from T. J. Richmond, (Fig. 1B; sequence in the first row) and designated as NCP146 in this study, was modified in order to introduce four *Eco72*I‐sensitive CpG dinucleotide sites (i.e., CACGTG) in the nucleosomal DNA. The constructed DNA is designated as CpG146 in this study (Fig. 1B; sequence in the second row). The DNA sequence of CpG146 is as follows: 5′‐ATCAA TATCC A**CG**TG CCAGT TATAC CAAAA GTGTA TTTGG AAACT CCTAA CTGAA AAGGC ATGTT CA**CG**T GAATT CA**CG**T GAACA TGCCT TTTCA GTTAG GAGTT TCCAA ATACA CTTTT GGTAT AACTG GCA**CG** TGGAT ATTGA T‐3′. In this sequence, the underlined nucleotides are the positions that were modified from the original α‐satellite sequence of NCP146, and the bold CpG dinucleotide sequences are the methylation target sites. Sixteen copies of the half unit of the palindromic CpG146 DNA sequence were tandemly subcloned into the plasmid pWMD01 19. Large‐scale preparation of the CpG146 nucleosomal DNA was performed essentially as previously described 20.

### *Eco72*I digestion assay with non‐, hemi‐ and full‐methyl DNAs

In order to examine the sensitivity of *Eco72*I toward hemi CpG‐methylated DNA, we prepared double‐stranded oligonucleotides containing 5‐methylcytosine (5mC) in one strand (i.e., hemimethylated DNA) or both strands (i.e., fully methylated DNA), as well as that without 5mC (i.e., nonmethylated DNA). The oligonucleotide sequences derived from CpG146 DNA are as follows: (a), 5′‐CAATA TCCA**C** GTGCC AGTTA TACCA AAAGT G‐3′; (b), 5′‐CACTT TTGGT ATAAC TGGCA **C**GTGG ATATT G‐3′; (c), 5′‐CTGAA AAGGC ATGTT CA**C**GT GAATT C‐3′; (d), 5′‐GAATT CA**C**GT GAACA TGCCT TTTCA G‐3′. The oligonucleotides (^m^a), (^m^b), (^m^c), and (^m^d) contain 5mC at the bold position of cytosine (C), respectively (Fig. 1C). For the preparation of the double‐stranded oligonucleotides, pairs of single‐stranded oligonucleotides (5 μm each) were annealed by heating at 95 °C for 5 min, followed by gradual cooling to 25 °C in 10 mm Tris‐HCl buffer (pH 8.0) containing 100 μm EDTA. The double‐stranded oligonucleotides were digested by *Eco72*I (Thermo Fisher Scientific; cat. ER0361) at 37 °C for 16 h with 1.8 units of *Eco72*I per pmol of DNA, in 1× Tango Buffer (33 mm Tris‐acetate buffer [pH 7.9] containing 10 mm magnesium acetate, 66 mm potassium acetate, and 100 μg·mL^−1^ BSA) from the manufacturer. The reaction samples were electrophoresed under denaturing conditions, using a 20% polyacrylamide gel containing 8 m urea.

### Purification of the *de novo* CpG methyltransferase *M.Sss*I

The cDNA encoding the full‐length *Spiroplasma M.Sss*I (Swiss‐Prot: P15840), with codons optimized for expression in *E. coli*, was custom‐synthesized by Eurofins Genomics (Tokyo, Japan). The cDNA was subcloned into pET15b at the *Nde*I and *Xho*I restriction digestion sites. For bacterial expression of the *M.Sss*I protein, T7 Express *lysY* Competent *E. coli* (High Efficiency) cells from NEB (cat. C3010I), harboring the pET15b‐*M.Sss*I plasmid, were grown at 37 °C in LB medium containing 100 μg·mL^−1^ ampicillin and 2% glucose. When the cells reached an OD_600_ of approximately 0.6, *M.Sss*I expression was induced by the addition of 50 μm IPTG, and the cells were further cultured at 16 °C for 16 h. Purification of the *M.Sss*I protein was performed essentially as described previously 34. We used a final dialysis buffer composed of 10 mm Tris‐HCl (pH 8.0) buffer, containing 500 mm NaCl, 2 mm DTT, 100 μm EDTA, and 50% glycerol. The specific activity of the purified *M.Sss*I enzyme was determined using CpG146 DNA as substrate, in comparison with the activity unit of the NEB *M.Sss*I enzyme (cat. M0226M), by quantifying the amounts of the *Eco72*I‐digested DNA fragments.

### Large‐scale methylation of CpG146 DNA and its verification

For the preparation of CpG‐methylated nucleosomal DNA, 50 μg·mL^−1^ of the CpG146 DNA was incubated at 37 °C for 16 h with *M.Sss*I (6 units·μg^−1^ DNA), in 10 mm Tris‐HCl buffer (pH 8.0), containing 50 mm NaCl, 2.5 mm EDTA, and 640 μm SAM. To separate the reacted nucleosomal DNA from the *M.Sss*I, the reaction was extracted with phenol–chloroform–isoamyl alcohol (25 : 24 : 1), and the DNA was ethanol‐precipitated. A small portion of the reacted DNA was digested with *Eco72*I (Thermo Fisher Scientific; cat. ER0361) or *Msp*JI (NEB, cat. R0661S), at 1.8 units·pmol^−1^ DNA and 0.45 units·pmol^−1^ DNA, respectively. The CpG‐methylated CpG146 DNA was also used in the binding assay with MBD2, using an EpiXplore methylated DNA enrichment kit (Takara Bio, Shiga, Japan; cat. 631963). In each MBD2‐binding reaction, the histidine‐tagged MBD2 protein (5 μg) from the kit, which was immobilized onto 4 μL of TALON resin, was incubated with 500 ng of the 146‐bp nucleosomal DNA at 25 °C for 1 h. The incubated samples were washed four times, using the 1× binding/washing buffer of the kit, and the nucleosomal DNAs were eluted with elution buffer (high). The nucleosome DNA of each fraction was ethanol‐precipitated and suspended in 20 μL of 10 mm Tris‐HCl buffer (pH 8.0) containing 100 μm EDTA. One quarter (5 μL) of each fraction was loaded onto a 5% polyacrylamide gel and analyzed by electrophoresis.

### Reconstitution of the CpG‐methylated NCP for crystal structure analysis

Reconstitution of the NCPs was performed by the salt gradient method, essentially as described previously 19, 20, using bacterially expressed, purified human histones H2A, H2B, H3, and H4 20, and *M.Sss*I‐treated or ‐untreated CpG146 DNA. The reconstituted NCPs were heat‐shifted at 42 °C for 1 h, and precipitated by the addition of MgCl_2_ to a final concentration of 12 mm. The reaction solutions were incubated for 15 min at 25 °C, and the NCPs were precipitated by centrifugation at 17 500 ***g*** for 10 min at 4 °C. Then, the precipitated NCPs were separated from the supernatant containing the free nucleosomal DNA. For crystallization, the precipitated NCPs were resolved in CCS buffer (20 mm potassium cacodylate buffer [pH 6.0] containing 1 mm ethylenediamine tetraacetic acid) 20. Crystallization was performed by the hanging‐drop vapor‐diffusion method at 20 °C. The drops were prepared by mixing 1.0 μL of potassium cacodylate buffer (pH 6.0), containing 85–110 mm manganese(II) chloride and 110–140 mm potassium chloride, with 1.0 μL of the NCP solution (4–6 mg·mL^−1^) in CCS buffer, and were equilibrated against 500 μL of the reservoir solution (20 mm potassium cacodylate buffer [pH 6.0], containing 45 mm manganese(II) chloride and 35 mm potassium chloride). Single crystals typically appeared within 2–7 days. The crystals were transferred to 20 mm potassium cacodylate buffer (pH 6.0), containing 37 mm manganese(II) chloride, 40 mm potassium chloride, 2% trehalose, and 24% MPD, and then flashed cooled with liquid nitrogen.

### Data collection, structure determination, and refinement

Diffraction data were collected at 100 K on the BL32XU beam line at SPring‐8 (Hyogo, Japan). Diffraction images were processed with XDS 35 and HKL2000 36. The structures were solved by molecular replacement (MR) with PHASER 37, using the structural coordinates of the 146‐bp DNA in NCP146 (PDB: 1KX3) 22 and the histone octamer in the human NCP (PDB: 2CV5) 38 as the search models. Structural refinement was accomplished with the PHENIX suite 39, and manual model building in Coot 40. pymol (The PyMOL Molecular Graphics System) was used to render the structural figures and for general manipulations. The final refinement statistics are summarized in Table 1.

### PDB accession numbers

The structural coordinates have been deposited in the Protein Data Bank, under the accession codes PDB: 5B2J (the CpG‐methylated CpG146 NCP) and PDB: 5B2I (the unmodified CpG146 NCP), respectively.

## Author contributions

YF performed the structural analysis. MW performed the biochemical analysis. TU and SY designed the research. All authors analyzed data and wrote the paper.
